# Quality of life of caregivers with relatives suffering from mental illness in Hong Kong: roles of caregiver characteristics, caregiving burdens, and satisfaction with psychiatric services

**DOI:** 10.1186/1477-7525-10-15

**Published:** 2012-01-31

**Authors:** Daniel Fu Keung Wong, Angus Yuk Kit Lam, Sau Kam Chan, Shuk Fan Chan

**Affiliations:** 1 Applied Social Studies, City University of Hong Kong, Hong Kong SAR; 2 Baptist Oi Kwan Social Service, Hong Kong SAR

**Keywords:** Quality of Life, Caregivers characteristics, Caregiving Burden, Satisfaction with psychiatric services, Hong Kong

## Abstract

**Background:**

The study attempted to explore the quality of life (QoL) of Chinese caregivers with mentally ill relatives. It also aimed to examine the differential roles of caregiving burdens, caregiver characteristics, and satisfaction with psychiatric services in caregivers' QoL.

**Methods:**

276 caregivers with relatives attending community psychiatric facilities in Hong Kong were invited to fill out a questionnaire. One sample t-tests were conducted to compare the results of this study with that of other Chinese populations in Hong Kong, Taiwan, and mainland China. A hierarchical regression analysis was performed to examine the relative influence of different factors on caregivers' QoL.

**Results:**

Our sample of caregivers had significantly lower QoL scores than other Chinese populations. Results also suggest that Chinese caregivers who had chronic illness, younger in age, a lower education level, experienced more difficulties in handling negative symptoms, and were more dissatisfied with mental health services had poorer quality of life. Indeed, caregiver characteristics displayed a much stronger association with caregivers' QoL than did caregiving burdens and satisfaction with psychiatric services.

**Conclusions:**

This study supports the strong association of caregiver characteristics and the QoL of caregivers and establishes the nature of the relationship between satisfaction with mental health services and caregiver QoL. Implications for future research and practice are discussed.

## Introduction

Caring for a family member with mental illness can be burdensome. Caregivers may experience financial burdens, difficulty handling disruptive behavior and fluctuating emotions that cannot be controlled, a lack of time for personal enjoyment and social engagement, difficulty handling the lack of motivation found in the mentally ill family member, and financial difficulties [1,2]. Evidence of family burdens is also found among caregivers with different ethnic backgrounds such as Chileans [3], Swedes [4], Germans [5], Hong Kong Chinese [6,7], and mainland Chinese [8]. The impact of caring burdens on the psychological wellbeing of caregivers has also been well documented, the results generally suggesting that caregivers suffer from poor psychological health and that some of them may even develop mental illness [2,6].

**Figure 1:**
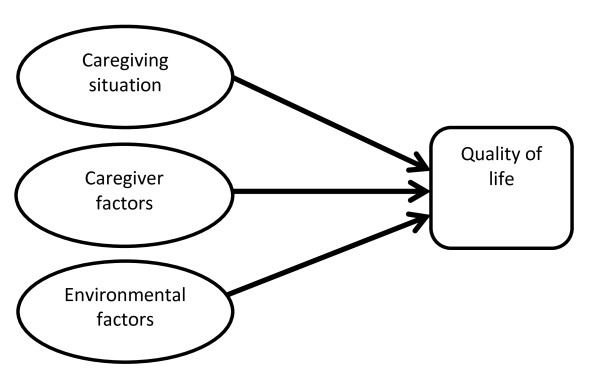
**A model with three sets of factors affecting the QoL of caregivers of family members with mental illness (adapted from the model of White et al., 2004 **[14]).

Although it is commonly acknowledged that caregiving burdens can lead to caregivers with a relative suffering from mental illness experiencing poor quality of life (QoL) [2], few studies have explored this relationship empirically. The few studies that have done so found that family burdens were inversely related to the QoL of caregivers with children suffering from mental health problems [9], mood disorders [10], and schizophrenia [4]. Similar studies have also been conducted on caregivers with different ethnic backgrounds, the findings also suggesting that Sudanese caregivers [11] and Kuwaiti caregivers [12] with greater caregiving burdens have poorer QoL. A careful review of the literature identified only one English language article concerning caregivers' burdens and the QoL of Chinese caregivers with a schizophrenic family member [13]. The results suggest that the best QoL predictors for such Chinese family caregivers are physical health and household income. However, the study in question is subject to a number of limitations. Because Chinese caregivers living in different parts of China and the world are exposed to different sets of societal and contextual factors influencing their caring experiences, the findings generated by Li et al. may not be applicable to Chinese people living in other parts of China and the world [13]. Second, because the samples considered in their study were taken from three hospitals, the caregivers who took part in the survey could have been experiencing a higher level of stress due to their relatives' hospitalization. Lastly, caregivers who are living with their mentally ill family members in the community may have different caregiving burden experiences that affect their QoL in different ways. To the best of our knowledge, no previous study has investigated the caregiving burdens and QoL of Chinese caregivers in Hong Kong. This study intended to explore the quality of life of a group of Chinese caregivers whose mentally ill relatives were living in the communities in Hong Kong; and to examine different factors that might influence caregivers' quality of life. In the literature, there are three sets of factors - (1) caregiving situation; (2) caregiver factors; and (3) environmental factors - that can potentially impact on the QoL of Chinese caregivers with relatives suffering from mental illness in Hong Kong (See Figure 1).

### Caregiving situation

The 'caregiving situation' can be defined as variables related to characteristics of the patient's illness that impact on the QoL of the caregiver. These factors may include the functional status of the patient or the caregiver's perception and evaluation of the care needs of the patient. In the mental health literature, objective and subjective burdens are commonly defined as caregiving situations which affect the QoL of caregivers with relatives suffering from mental illness. The 'objective burden' may be defined as 'all those things that the caregiver and/or his or her family has to do (e.g. helping and supervising), experiences (e.g. disturbed family and/or social relations), or is not allowed to do (e.g. hobbies and work) as a consequence of caregiving [14]. It also includes the tasks of handling the emotional needs of the mentally ill relative [2]. On the other hand, the 'subjective burden' may be defined as the distress experienced by the caregiver in dealing with the objective burden [15], potentially including feeling trapped, guilty, shameful, isolated, and emotionally exhausted [15]. Studies in the literature have suggested that Chinese caregivers (i) experience difficulties managing the negative symptoms presented by relatives with mental illness and handling positive symptoms [6]; (ii) pay high psychological and social costs when caring for their relatives [6]; and (iii) have poor mental health outcomes [6,7]. However, no study has examined the caregiving burdens and QoL of Chinese caregivers in Hong Kong. In this study, 'caregiving burdens' are defined as perceived strains associated with handling positive and negative symptoms presented by mentally ill relatives and the social and psychological costs borne when caring for such relatives.

### Caregiver factors

According to White et al. [16], caregiver factors are conceptualized as characteristics of the caregiver - who he/she is (i.e. age, gender, etc.) and his/her personal attributes - that may impact on the caregiver's QoL. Sales [2] argued that many studies fail to examine contextual variables - such as life circumstances and demographic characteristics - that may strongly affect the QoL of caregivers with family member suffering from mental and other chronic illnesses. These demographic characteristics may include the socioeconomic status, ethnicity, age, gender, and family stage and composition of the caregiver. For example, Awadalla et al. [11] found that among caregivers of Sudanese psychiatric patients, those who were females, parents, less educated, older, or had poor physical health were associated with poor QoL. In China, Li, Lambert & Lambert [13] revealed that among caregivers with schizophrenic relatives, those who had higher household income and better physical health had better QoL. However, because of differences in societal environments, there is uncertainty as to whether the findings of Li et al.'s study are applicable to Chinese populations outside mainland China.

### Environmental factors

Environmental factors include support from family and friends, support from the health care system, and the availability of and satisfaction with environmental resources [16]. Marsh and Johnson [17] maintained that there is a need to pay greater attention to the difficulties caregivers face when dealing with the health care system because caregivers' efforts can place them under tremendous strain and potentially impact on their QoL. However, a review of the literature showed very few empirical studies exploring the impact of environmental factors such as satisfaction with services on the QoL of caregivers with relatives suffering from mental illness. In this study, we attempted to examine the relationship such environmental factors have with QoL among caregivers with relatives suffering from mental illness.

Since previous findings appear to suggest that caregiving burdens are consistently related to poor mental health and QoL among caregivers, one assumption made in this study was that such burdens would have the strongest association with the QoL of caregivers. Our study also hypothesized that caregiver factors and environmental factors are the second and third most important sets of factors associating with the QoL of caregivers. This prioritization was based on the findings relating to each such set of factors reflected in the literature.

### Objectives

This study aimed at explore the quality of life and caregiving burdens of caregivers with relatives suffering from mental illness in Hong Kong; and examining the association between caregiving burdens, caregiver characteristics, satisfaction with mental health services and the QoL of caregivers.

### Hypotheses

There were three hypotheses in this study: (1) caregivers with greater caregiving burdens have poorer quality of life; (2) caregivers with a lower level of satisfaction with mental health services have poorer quality of life; and (3) 'caregiving burdens' had the stronger association with the QoL of caregivers than do 'caregiver factors' and 'satisfaction with psychiatric services'.

## Method

### Participants

'Caregiver' in this study refers to a person with a mentally ill family member. It is not necessary that the caregiver live with the mentally ill family member, but they have to provide regular support, such as visits and daily care, to the ill relative. 'Ill relative' refers to a person with mental illness. The inclusion criteria were: caregivers (i) aged 18 or above; (ii) able to speak and write simple Chinese; (iii) who provided regular support for a mentally ill relative.

### Research design and sampling procedures

A cross-sectional survey design and a convenience sampling method were adopted for this study. This sampling method was chosen because of the absence of a list of caregivers in Hong Kong and the greater feasibility of obtaining a sample of caregivers through various social service organizations in all 18 Hong Kong districts. Participants were caregivers of individuals with mental illness who were members of the various community-based mental health service units. The nature of these service units were half-way houses, integrated community mental health services and social clubs. The research team approached the caregivers and gave details of the survey to them. After signing the consent form, the participants were given a self-administered questionnaire. Since some participants were in their old age and might also be illiterate, the research team provided special assistance to facilitate the completion of their questionnaire. The participants were assured of the voluntary nature of the survey and of the fact that declining to participate in the survey would have no adverse effect on their use of services. It took around 35 minutes to complete the questionnaire. This study was endorsed by the Research Ethics Committee of the Baptist Oi Kwan Social Services Organization in Hong Kong, which sponsored the project.

### Instruments

The questionnaire contained socio-demographic questions and the following instruments:

#### Perceived Chronic Strains Scale (Short Form)

This is a four-point scale denoting the severity of perceived strains experienced by caregivers in their day-to-day care of their mentally ill relatives in which the responses range from 'Not stressful at all' (1) to 'Very stressful' (4). The original scale consisted of 32 items categorized into four subscales: (i) difficulty managing drug compliance and follow-up; (ii) difficulty managing bizarre and disturbing behavior among individuals with mental illness; (iii) difficulty handling negative symptoms of persons with mental illness; and (iv) social costs associated with the constant care of people with mental illness [6]. The short form of the scale employed in the study had only 16 items. A test of reliability revealed an acceptable level of internal consistency (Cronbach's alpha = 0.933).

#### Satisfaction with Mental Health Services in Hong Kong

The research team developed this scale to evaluate caregivers' satisfaction with 10 community-based mental health services available to their mentally ill relatives in Hong Kong, such as specialist outpatient clinics and supported employment. This five-point scale ranged from 'Very dissatisfied' (1) to 'Very satisfied' (5), and the caregivers were encouraged to rate only those services that their relatives had used or were using. Examples of questions asked are: were the caregivers satisfied with (i) the opening hours for the services; (ii) the services provided by psychiatrists, nurses, etc.; and (iii) the amount they had to pay? A test of reliability revealed an acceptable level of internal consistency (Cronbach's alpha = 0.867 to 0.947).

#### World Health Organization Quality of Life Scale - BRIEF Version (HK)

The World Health Organization Quality of Life Scale - Brief Version is a 26-item, self-administered questionnaire [18]. Subjects assess their satisfaction with each item in the past 2 weeks on a 5-point scale (from 1 = very dissatisfied to 5 = very satisfied). The 26 items can be divided into four subscales: physical health, psychological health, social relationships, and environmental factors. The scale has well-established psychometric properties and has been widely used in different cultures. The Cronbach's alpha values for this scale and its subscales ranged from 0.684 to 0.810, indicating an acceptable level of internal consistency.

## Results

Table 1 shows the demographic profile of the participants. The data were drawn from throughout the territory and covered all 18 administrative districts of Hong Kong. Most of the caregivers who participated in this study were mothers and/or females. The typical age of the caregivers was relatively high, with over 70% being more than 51 years old. In terms of employment status, the largest groups were housewives and retirees (39.7% and 24.1%, respectively). About 58% had monthly family income of between HK$5000 and HK$20000 (US$1 = HK$7.80). Thirty-six percent of the ill relatives suffered from schizophrenia, while 18.6% had been diagnosed with early psychosis.

**Table 1:** Demographic characteristics of caregivers (n = 276)

Characteristic	%
Sex	
Male	20.1
Female	79.9
Age	
20-40	8.8
41-50	16.8
51-60	37.2
61-above	37.2
Marital status	
Single	8.2
Married	74.5
Divorced	4.8
Widowed	12.5
Education	
Primary or below	41.5
Secondary	45.5
University	13.0
Employment	
Full-time	23.3
Housewife	39.7
Student	1.2
Part-time	8.2
Unemployed	3.5
Retired	24.1
Income	
Social security scheme	9.2
5000-20000	57.9
20001-40000	17.6
40001 or above	6.6
Unknown	8.7
Relationship	
Father	12.0
Mother	57.1
Child	8.7
Sibling	10.9
Spouse	11.3
Years of care	
0-5	29.1
6-10	26.2
11-20	26.9
Above 20	17.8
Type of mental illness of the family member	
Schizophrenia	36.1
Early psychosis	18.6
Delusion	5.4
Bipolar affective disorder	12.2
Depression	13.1
Anxiety disorders	12.3
Others	2.2
Living together	

Table 2 displays the caregiving strains experienced by the caregivers. The most highly rated strains experienced by the caregivers were related to 'difficulty handling bizarre and disturbing behavior' and 'difficulty managing fluctuating emotions of ill relatives' (M = 2.96, M = 2.96). Next on the list of caregiver strains were 'difficulty handling suicidal thoughts/attempts committed by the ill relative' and the issue of the ill relative's lack of employment (M = 2.83, M = 2.78).

**Table 2:** Caregiving burdens of caregivers

	Mean
1. Bizarre behaviour manifested by the ill relative (e.g. muttering to oneself)	2.96
2. Fluctuating emotions of the ill relative○	2.96
3. Suicidal thoughts or attempts made by ill relative	2.83
4. Being unemployed	2.78
5. Ill relative refuses to take medication	2.72
6. Disputes among family members arising from differences on how to handle the ill relative	2.72
7. Destructive behaviour shown by ill relative (e.g. damaging furniture)	2.72
8. Ill relative refuses to go for medical follow-ups	2.69
9. Household life affected due to care of the person	2.63
10. Financial difficulties because of having to take care of the ill relative	2.58
11. Ill relative neglects personal hygiene	2.57
12. Ill relative refuses to go to work	2.56
13. Ill relative spends a great deal of time in bed	2.55
14. Ill relative idle at home	2.47
15. Cannot participate in social activities due to care of the ill relative	2.31
16. Ill relative refuses to perform household chores	2.16
Overall mean	2.09

**Figure 2:**
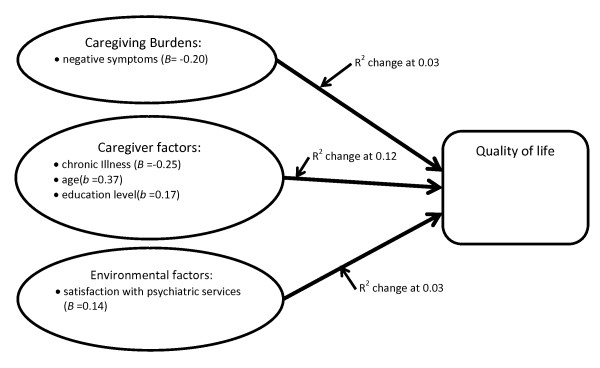
**Impact of specific variables affecting QoL of caregivers of family members with mental illness**.

Hierarchical regression analysis was performed to test the relative impacts of the three sets of factors (i.e. caregiving situation, caregiver characteristics, and environmental factors) on the caregivers' QoL. In step 1, the caregiving situation (i.e. caregiving burdens) - including 'difficulty handling bizarre behavior and excessive and uncontrollable emotions', 'difficulty handling negative symptoms', and 'social costs associated with the care of a relative with mental illness' - was entered into the first block. Caregiver characteristics such as the year in which the caregiver assumed responsibility for the relative's care, the caregiver's own chronic illness, family income, and the age of the caregiver were entered as the second block. The final block consisted of the environmental factor of 'satisfaction with mental health services'. The results suggest that the Chinese caregivers who had chronic illness, younger in age and a lower education level had poorer quality of life. As a domain, caregiver characteristics explained 12% of the variance in QoL of Chinese caregivers with relatives suffering from mental illness in Hong Kong (Table 3), (see Figure 2). 'Caregiving burdens' and 'satisfaction with mental health services' also displayed significant association with caregiver's QoL with explained variances of 3%. Of the three subscales for chronic strains, only 'difficulty handling negative symptoms' had significant association with the QoL of the caregivers. In other words, Chinese caregivers who experienced more difficulties in handling negative symptoms and were dissatisfied with mental health services had poorer quality of life. To conclude, the results supported hypothesis 1 and hypothesis 2, the hypothesis 3 was rejected.

**Table 3:** Differential impact of caregiving situation, caregiver characteristics, and environmental factors in influencing QoL of caregivers

Predictors	Beta	***R***^**2**^	*R*^2 ^change	*F *change
Step 1				
Caregiving situation (Chronic strains)		0.03	0.03	2.53
Difficulty handling bizarre behavior and excessive and uncontrollable emotions	-0.06			
Difficulty handling negative symptoms	-0.20*			
Social costs associated with care	-0.13			
Step 2				
Caregivers' characteristics		0.15	0.12	4.78
Year assumed responsibility for care of the relative	-0.05			
Caregiver's own chronic illness	-0.25***			
Family income	0.01			
Living together with the relative	-0.02			
Age of caregiver	0.31***			
Caregiver's education level	0.17*			
Step 3				
Environmental factors (Satisfaction with mental health services)	0.14*	0.18	0.03	5.31

* < 0.05, ** < 0.01, *** < 0.001

## Discussion

In this study, the quality of life of our caregivers was measured by The World Health Organization Quality of Life Scale - BRIEF Version, which has been used to measure the quality of life of other Chinese populations in Taiwan and mainland China [13,18,19]. With the availability of data in the other studies, one sample t-tests were performed to examine the differences in QoL scores between Chinese caregivers in the three places [Table 4]. The results indicate that that the caregivers in our sample had significantly poorer QoL than the general public in Hong Kong [18], mainland China [13], and Taiwan [19], and that their QoL was significantly lower than that of their caregiver counterparts in mainland China [13]. Some of these results should not be surprising, because numerous studies have already found that caregivers of relatives with special needs have lower QoL than the general population [20,21]. Nevertheless, what is surprising is that our group of caregivers had poorer QoL than caregivers in mainland China. Different expectations on the availability and adequacy of psychiatric services may explain the QoL difference between the two groups. In mainland China, very few community-based psychiatric services are available in the community, and members of the general public are largely expected to care for and support mentally ill relatives themselves. In contrast, there is a high expectation that the government will provide adequate psychiatric services in Hong Kong that invariably heightens caregivers' and consumers' expectations and sometimes leads to dissatisfaction with government involvement in the provision of psychiatric services. Indeed, the government of Hong Kong Special Administrative Region has openly acknowledged that it will uphold the Chinese family tradition of caring for the ill relative, and that it would support these families to take care of the relative who is suffering from a mental illness to live in the community [22]. This difference in people's expectations of the government's role in relation to their own responsibility for caring for ill relatives might affect how they evaluate their own well-being and thus explain the difference in QoL scores between caregivers in Hong Kong and mainland China.

**Table 4:** A comparison table of QoL scores between our sample of caregivers and those found in Taiwan and China

*Our sample versus caregivers of relatives with schizophrenia in China (n = 96)*				
	**Our sample**	**Caregivers in China**	***t ***	***p***

QOL Physical	13.37(2.40)	14.08(2.36)	-4.86	< 0.001
QOL Psychological	12.07(2.55)	12.92(2.68)	-5.51	< 0.001
QOL Social	12.87(2.36)	14.44(2.40)	-11.09	< 0.001
QOL Environmental	12.61(2.10)	12.32(2.60)	2.29	0.023

*Our sample versus Hong Kong general population (n = 155)*				

	Our sample	HK general population		

QOL Physical	13.37(2.40)	15.85(2.13)	-17.12	< 0.001
QOL Psychological	12.07(2.55)	14.77(2.39)	-17.56	< 0.001
QOL Social	12.87(2.36)	14.26(2.39)	-9.82	< 0.001
QOL Environmental	12.61(2.10)	13.74(2.45)	-8.96	< 0.001

*Our sample versus the general population in China (n = 50)*				

	Our sample	China general population N = 50	*t*	*p*

QOL Physical	13.37(2.40)	15.8(2.9)	-16.771	< 0.000
QOL Psychological	12.07(2.55)	14.3(2.5)	-14.49	< 0.000
QOL Social	12.87(2.36)	13.7(3.0)	-5.87	< 0.000
QOL Environmental	12.61(2.10)	13.2(2.4)	-4.68	< 0.000

One sample t-test *p *< .05				

*Our sample versus the Taiwanese general population (n = 132,045)*				

	Our sample	Taiwan general population	*t*	*p*

QOL Physical	13.37(2.40)	15.05(2.08)	-11.58	< 0.000
QOL Psychological	12.07(2.55)	13.61(2.27)	-10.00	< 0.000
QOL Social	12.87(2.36)	14.39(2.20)	-10.74	< 0.000
QOL Environmental	12.61(2.10)	13.00(2.15)	-3.09	< 0.002

A major objective of this study was to test the relative influences of different sets of factors on the QoL of caregivers with relatives suffering from mental illness. The results indicate that caregivers' characteristics exerted the greatest influence on the QoL of the caregivers, with a total explained variance of 12%. In particular, the caregiver's age, education level, and chronic illness had significant impacts on their QoL. These findings are different from those hypothesized, but serve to support Sales' assertion that "the contextual variables - such as life circumstances and demographic characteristics - may strongly affect the QoL of caregivers with [a] family member with chronic illness (including mental illness)" [2]. On the other hand, although caregiving burdens can and do affect the QoL of caregivers, their influence might subside with the passage of time, with some caregivers becoming more adjusted to their caregiving role and acquiring more skills in managing mentally ill relatives. Consequently, they may come to experience or evaluate their caregiving role as less burdensome.

On the other hand, caregiver characteristics are more stable characteristics that are less amenable to change. Indeed, some of these characteristics are unchangeable (e.g. age), and others may follow a deteriorating course (e.g. chronic illness). The literature shows that caregiver characteristics affect the QoL of caregivers in two ways: through their direct and indirect effects. In the case of direct effects, caregiver characteristics affect caregivers' QoL irrespective of the caregiving burdens they experience. For instance, more highly educated caregivers tend to have better jobs, higher salaries, and more resources that can enhance their QoL [11]. However, these characteristics may interact with other factors such as caregiving burdens to exert indirect effects on the QoL of caregivers. For example, more highly educated caregivers may be able to use their financial and social resources to help them deal with their caregiving burdens, resulting in better QoL. Indeed, further statistical analysis of our data revealed such a result, showing a significant moderating effect of the interaction between caregiver's education level and caregiving burdens on the QoL of caregivers (*B *= 0.309, *F *change = 2.80, *p *= 0.05, with 1% explained variance). Given the limited availability of evidence concerning the relationship between the characteristics and QoL of caregivers with mentally ill relatives [16], future studies should provide more empirical data to clarify the nature of this relationship.

The second set of variables - caregiver's strains - explained 3% of the variance in the QoL of caregivers. Further analysis showed that only negative symptoms had a significant impact on the QoL of caregivers who participated in this study. These findings echo those findings found in a previous study in Hong Kong [6] and overseas [23]. One possible explanation for these findings is that although positive symptoms may be difficult to manage, they may be perceived as 'part of the illness' beyond the caregiver's control and therefore as symptoms that have to be tolerated and accepted. On the other hand, caregivers may perceive negative symptoms such as 'idle at home' and 'refuse to go to work' as forms of 'laziness' that are amenable to change through personal willpower. This attitude may in fact be rooted in the Chinese belief in Confucian work ethics emphasizing hard work, perseverance, and patience. Simply put, in Chinese culture, there is a strong focus on one's productivity, and anyone who does not work and remains at home is likely to be considered lazy and unproductive [24]. Thus, it was not surprising to find that some caregivers might have had difficulty accepting the negative symptoms presented by their ill relatives. Moreover, unlike positive symptoms which may be more amenable to medication, negative symptoms are issues caregivers have to spend time and energy addressing every day. Further studies are needed to clarify whether the above assumptions are correct and to develop strategies on raising Chinese caregivers' awareness of how such attribution may affect their caregiving burdens and quality of life. At present, there is only one government subsidized family resource centre for family members with relatives suffering from mental illness in Hong Kong. However, many community mental health services are also providing psycho-education programmes for family members. It would be interesting to incorporate the above-mentioned cultural values into the psycho-education programmes, and to examine whether an increase in such awareness might reduce the culturally biased perception of negative symptoms found among some Chinese family members with relatives suffering from mental illness.

Turning to environmental factors, satisfaction with mental health services explained 3% of the variance in the QoL of the caregivers. As noted in the literature review, very few studies have examined the relationship between satisfaction with psychiatric services and caregivers' quality of life. Our study makes a contribution to the literature by providing initial evidence supporting the existence of such a relationship. However, further and larger-scale research should be conducted to provide more concrete data specifying the nature of the relationship between these two variables. Moreover, in the Hong Kong context, studies should also evaluate how certain mental health services do or do not meet the needs of caregivers. This research should have a particular focus on developing our understanding of caregivers' knowledge of the different types of services available and the adequacy and accessibility of such services. The knowledge generated would facilitate the future development of policies and programs encouraging caregivers to utilize formal psychiatric services to their full extent.

### Limitations

This study has a number of limitations. First, although our sample of caregivers came from different districts in Hong Kong, they were not randomly selected. Therefore, the results cannot be generalized to the wider population of Hong Kong caregivers of mentally ill relatives. Second, our *Satisfaction with Mental Health Services Scale *was self-constructed, and further and more vigorous validation of the scale is needed. Third, there may be other variables such as social support and certain personality attributes in the three domains that influence the QoL of caregivers in Hong Kong. Researchers should consider including these variables in future studies.

## Conclusion

This study had attempted to clarify the roles of a set of factors associated with the QoL of Chinese caregivers in Hong Kong. It is found that caregivers who were younger, had chronic illness, a lower level of education (caregiver's characteristics), experienced more caregiving burdens and had more dissatisfaction with mental health services had poorer QoL. In particular, caregiver characteristics appeared to have the strongest association with caregiver's QoL. Future research should adopt a longitudinal study design to examine the relationship between QoL and these and other selected variables in the caregiver characteristic, caregiving situation and environmental factor domains, and should examine and impact of these variables in influencing the QoL of Chinese caregivers with relatives suffering from mental illness.

## Competing interests

The authors declare that they have no competing interests.

## Authors' contributions

DFKW is the person who has written the manuscript. AYKL was the coordinator of this research and had done the initial data analysis. SKC and SFC are staff of the agencies who had coordinated the data collection of this research and contributed to the discussion section of this manuscript. All authors read and approved the final manuscript.

## Authors' information

Daniel Fu Keung Wong^1 ^is professor of Department of Applied Social Studies, City University of Hong Kong, Hong Kong SAR. Mr. Angus, Yuk Kit Lam^1 ^is a research fellow of Department of Applied Social Studies, City University of Hong Kong, Hong Kong SAR. Miss Sau Kam Chan^2 ^is the Senior Service Coordinator of Baptist Oi Kwan Social Service, Hong Kong SAR, and Miss Shuk Fan Chan^2 ^is in charge of the Resource and Service Centre for the Relative of Ex-Mentally ill People, Baptist Oi Kwan Social Service, Hong Kong SAR.
